# Serum heme oxygenase-1 as a prognostic biomarker in patients with acute exacerbation of interstitial lung disease

**DOI:** 10.1038/s41598-023-49342-4

**Published:** 2023-12-19

**Authors:** Yoichi Tagami, Yu Hara, Kota Murohashi, Ryo Nagasawa, Hiroaki Fujii, Ami Izawa, Aya Yabe, Yusuke Saigusa, Miyu Kobayashi, Masafumi Shiida, Momo Hirata, Yukiko Otsu, Keisuke Watanabe, Nobuyuki Horita, Nobuaki Kobayashi, Takeshi Kaneko

**Affiliations:** 1 Department of Pulmonology, Yokohama City University Graduate School of Medicine, 3-9 Fukuura, Kanazawa-Ku, Yokohama, Kanagawa 236-0004 Japan; 2 Department of Biostatistics, Yokohama City University Graduate School of Medicine, Yokohama, Japan; 3 Research and Development Division, Minaris Medical Co., Ltd, 600-1 Minami-Ishiki, Nagaizumi-Cho, Sunto-Gun, Shizuoka, 411-0932 Japan

**Keywords:** Medical research, Biomarkers, Prognostic markers

## Abstract

Serum heme oxygenase (HO)-1 level has been reported as a clinically reliable diagnostic biomarker for acute exacerbation of interstitial lung disease (ILD); however, its utility for predicting mortality among these patients is unclear. Serum HO-1 levels of patients newly diagnosed with acute exacerbation of ILD were measured at the time of initiating steroid pulse therapy. The relationship between serum HO-1 and various other serum biomarkers, change in HRCT findings, and disease prognosis at 12 weeks after diagnosis of acute exacerbation was evaluated in 51 patients, of whom 17 (33%) had idiopathic pulmonary fibrosis (IPF). Serum HO-1 was higher in patients with acute exacerbation of IPF than in patients with acute exacerbation of other ILDs. Serum HO-1 levels were higher in patients who died within these 12 weeks than in survivors. Among age, sex, comorbidities, IPF diagnosis, HRCT findings, and blood biomarkers, serum HO-1 was a primary predictor of 12-week mortality. In 41 patients who underwent repeat HRCT, serum HO-1 was higher in patients with honeycomb progression than in those without. Serum HO-1 measurement could be useful for evaluating disease mortality and morbidity of patients with acute exacerbation of ILDs.

## Introduction

Interstitial lung diseases (ILDs) comprise a group of lung disorders characterized by various levels of inflammation and fibrosis. The disease activity, clinical course, and long-term prognosis are diverse among patients because of the wide range of pathological patterns including usual interstitial pneumonia (UIP), non-specific interstitial pneumonia, diffuse alveolar damage, organizing pneumonia, desquamative interstitial pneumonia, respiratory bronchiolitis, and their combinations. Acute exacerbation is recognized as a life-threatening condition with acute respiratory worsening with the typical histological pattern of diffuse alveolar damage superimposed upon lung fibrosis in various kind of ILD subtypes^1^. Among patients with acute exacerbation of idiopathic pulmonary fibrosis (IPF), the in-hospital mortality rate is reported to be in more than 50%^2^. A retrospective cohort study for acute exacerbation patients with non-IPF and IPF showed overall survival rates of 67% at 30 days, 43% at 60 days, and 40% at 90 days^3^. Other reports have shown mortality rates for acute exacerbation of connective tissue disease-associated ILD (CTD-ILD) ranging from 34 to 83%^4^. The prognostic variability of acute exacerbation seems to be influenced by various kinds of clinical conditions such as sex, age, pathological patterns including not only of diffuse alveolar damage, but also organizing pneumonia, pulmonary thromboembolism, diffuse alveolar hemorrhage, and bronchopneumonia, and comorbidities^5–7^. It is very difficult to predict acute exacerbation mortality and there are no established biomarkers for evaluating disease severity in these patients.

Oxidative stress which might play a role in disease progression among patients with ILD is caused by an imbalance between cellular production of reactive oxygen species and endogenous antioxidants such as classic antioxidant enzymes (catalase, glutathione peroxidase, and superoxide dismutase) and stress response protein (heme oxygenase (HO)-1)^8–11^. Much progress has been made in our understanding of the role of the classic antioxidant enzymes in mediating the lung's resistance against oxidant lung injury, and, it is becoming clear that other oxidant-induced gene products may also play vital roles in the lung's adaptive and/or protective response to oxidative stress. Several evidence has been reported aimed at elucidating the correlation between the fluctuation of these classic antioxidant enzymes and disease progression in lung cancer, obstructive pulmonary disease, and lung fibrosis, however this has not been put to practical use in clinical practice^12–17^. HO-1 known as stress response protein is a 32-kDa heat shock protein that converts heme into carbon monoxide, iron, and bilirubin, and is expressed exclusively on the anti-inflammatory M2 macrophage under oxidative stress condition^18^. Mumby et al. reported that HO-1 protein were highly upregulated in lung tissue and bronchoalveolar fluid with acute respiratory distress syndrome (ARDS) patients, reflecting the changes in iron mobilisation, signalling, and regulation^19^. Lakari et al. demonstrated that HO-1 immunoreactivity was detectable in alveolar macrophages of UIP and desquamative interstitial pneumonia, and also in the granulomas of pulmonary sarcoidosis^20^. Also, we have recently reported the utility of HO-1 in peripheral blood as an accurate biomarker of a diagnosis of acute exacerbation among patients with ILDs^21,22^. However, it is unclear whether serum HO-1 measurement could serve as a reliable biomarker for predicting disease prognosis among these patients.

The purpose of this study was to evaluate the ability of serum HO-1 level for predicting prognosis in patients with acute exacerbation of ILDs and discuss the mechanism by which elevated levels affect prognosis.

## Results

### Patient characteristics

**Figure 1:**
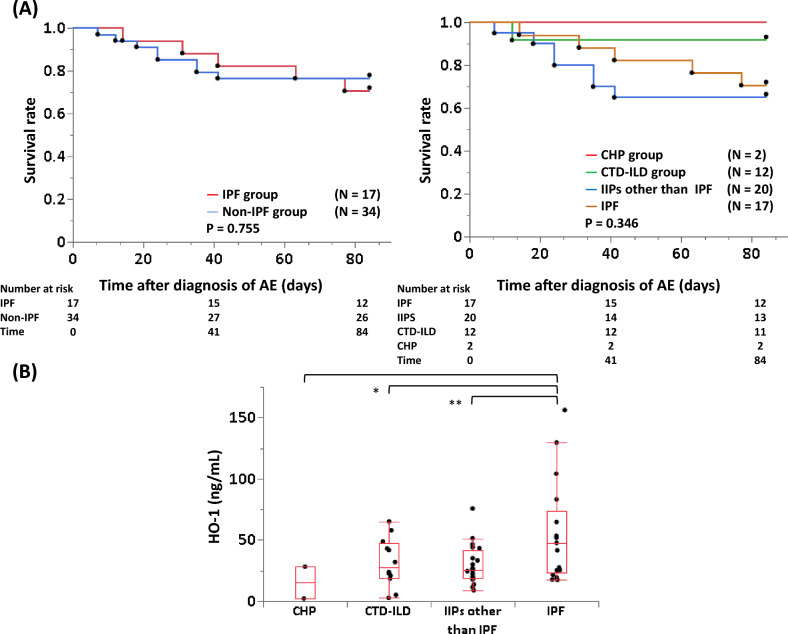
Comparison of survival curves (**A**) and serum heme oxygenase (HO)-1 (**B**) between acute exacerbation of idiopathic pulmonary fibrosis (IPF) vs. of non-IPF. (**A**) Comparison of the curves by log-rank test between acute exacerbation of IPF and non-IPF patients. (**B**) The median serum HO-1 levels for acute exacerbation of IPF, idiopathic interstitial pneumonias other than IPF and connective tissue disease-associated interstitial lung disease, and chronic hypersensitivity pneumonia were 47.6 [23.4–74.0], 25.7 [18.9–41.2], 27.9 [19.1–47.3], and 15.2 [2.2–28.2] ng/mL, respectively. The center bold line is the median value; the bottom and top of the boxes represent the 25th to 75th percentiles, respectively; and the whiskers are 95% confident intervals. *P < 0.05, **P < 0.01.

The clinical characteristics of 51 patients with acute exacerbation of ILD are summarized in Table 1. The median age was 75 [70–80] year and 38 (75%) patients were male. The enrolled patients were divided into 38 survivors and 13 non-survivors within 12 weeks after diagnosis of acute exacerbation. The median serum HO-1 level of the overall patients was higher than that in the normal control subjects (the overall patients, 27.5 [19.9–48.8] ng/mL vs. 2.9 [0.2–5.9] ng/mL, *P* < 0.001); the age matched subjects, 33.5 [19.9–92.7] ng/mL (65.5 year (n = 8)) vs. 1.5 [0.0 -5.2] ng/mL (65.5 year (n = 8)), *P* = 0.001). Also, the median serum HO-1 levels were significantly higher in non-survivors than in survivors (43.2 [25.7–79.5] ng/mL vs. 25.4 [18.5–44.7] ng/mL, *P* = 0.023). The underlying ILDs were IPF (n = 17), idiopathic interstitial pneumonias (IIPs) other than IPF (n = 20), CTD-ILD (n = 12), and chronic hypersensitivity pneumonia (n = 2). Comparison of survival curves between acute exacerbation in IPF and non-IPF patients by log-rank test showed no significant difference (*P* = 0.755, Fig. 1A). The median serum HO-1 in acute exacerbation of IPF was significantly higher than that in IIPs other than IPF and CTD-ILD (Fig. 1B).

**Table 1:** Patients’ characteristics.

Characteristic	Survivors (n = 38)	Non-survivors (n = 13)	Total patients (n = 51)	*P* value (Sv. vs. Non-sv. )
Age, y	74 (70–79)	79 (75–83)	75 (70–80)	0.069
Male sex	28 (74)	10 (77)	38 (75)	0.817
CCIS	3 (2–4.3)	3 (1.5–4)	3 (2–4)	0.597
Aetiology of acute exacerbation				0.476
Unknown	34 (89)	13 (100)	47 (92)	
Infection	1 (3)	0 (0)	1 (2)	
Drug	3 (8)	0 (0)	3 (6)	
Diagnosis of ILDs				0.304
IPF	12 (32)	5 (38)	17 (33)	
IIPs other than IPF	13 (34)	7 (54)	20 (39)	
CTD-ILD	11 (29)	1 (8)	12 (24)	
CHP	2 (5)	0 (0)	2 (4)	
Blood biomarkers				
P/F ratio	244 (209–331)	215 (148–383)	239 (183–331)	0.456
Serum LDH, U/L	274 (217–358)	336 (253–413)	275 (233–373)	0.353
Serum KL-6, U/L	1066 (653–1751)	406 (285–1056)	938 (403–1600)	0.029
Serum SP-D, ng/mL	221 (120–313)	203 (63–322)	216 (105–310)	0.329
Serum HO-1, ng/mL	25.4 (18.5–44.7)	43.2 (25.7–79.5)	27.5 (19.9–48.8)	0.023
HRCT score (baseline)				
GGO score	8.5 (6–11)	10 (8–15.5)	11 (7–11)	0.181
Honeycomb score	3.5 (1–8)	4 (3–8)	4 (1–8)	0.610
HRCT score (repeat)				
GGO score	6 (4–7)	9 (6.8–17)	6 (4–7)	0.008
Honeycomb score	6 (2–10)	6 (3.5–11.3)	6 (2–10)	0.753
Treatment				
Corticosteroid pulse therapy	12 (92)	33 (87)	45 (88)	0.598
Pulse frequency	1 (1)	1 (1–2)	1 (1–2)	0.110
NEI use	6 (16)	5 (38)	11 (22)	0.086
Outcome				< 0.001
3-month mortality	0 (0)	11 (85)	11 (22)	
BSC	0 (0)	2 (15)	2 (4)	

Data are presented as the median (25th–75th percentiles) or the number (%).

BSC, best supportive care; CCIS, Charlson Comorbidity Index score; CHP, chronic hypersensitivity pneumonia; CTD, connective tissue disease; GGO, ground-glass opacity; HO-1, heme oxygenase-1; HRCT, high-resolution computed tomography; IIPs, idiopathic interstitial pneumonias; ILDs, interstitial lung diseases; IP, interstitial pneumonia; IPF, idiopathic pulmonary fibrosis; KL-6, Krebs von den Lungen-6; LDH, lactate dehydrogenase; NEI, neutrophil elastase inhibitor; P/F ratio, partial pressure of oxygen in arterial blood/fraction of inspiratory oxygen; SP-D, surfactant protein-D.

### Relationship between serum HO-1 and other clinical parameters

**Figure 2:**
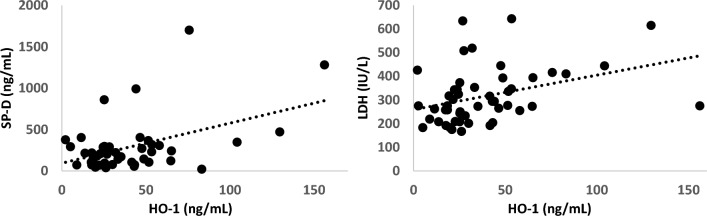
Relationship of serum heme oxygenase (HO)-1 with surfactant protein (SP)-D and lactate dehydrogenase (LDH). Serum HO-1 correlated positively with serum SP-D and LDH (*P* = 0.46 and *P* = 0.37, respectively).

As shown in Fig. 2, serum HO-1 correlated positively with serum surfactant protein (SP)-D and lactate dehydrogenase (LDH) (r = 0.46 and r = 0.37, respectively). Correlation coefficient between serum HO-1 with any other clinical parameter (age, Charlson Comorbidity Index score [CCIS], partial pressure of oxygen in arterial blood/fraction of inspiratory oxygen [P/F ratio], serum Krebs von den Lungen [KL]-6) or with baseline HRCT parameters of ground-glass opacity (GGO) and honeycomb scores was low^23,24^.

### ROC curve analysis

Table 2 summarizes the results of receiver-operating characteristic (ROC) curve analysis of the ability of serum HO-1 level and other parameters to predict 12-week mortality among patients with acute exacerbation. Area under the ROC curve (AUC) was relatively high for serum HO-1 level and GGO score (0.71 and 0.63, respectively). For serum HO-1, the best cut-off level was 41.8 ng/mL, which had sensitivity of 62% and specificity of 74% for predicting 12-week mortality.

**Table 2:** ROC curve analyses for the serum HO-1 level and other parameters to predict 3-month mortality among patients with acute exacerbation.

Variable	N	AUC	Best cut-off value	Sensitivity, %	Specificity, %	P value
Serum HO-1, ng/mL	51	0.71	41.8	62	74	0.016
P/F ratio	39	0.58	198	50	78	0.793
Serum LDH, U/L	51	0.59	336	54	71	0.446
Serum SP-D, ng/mL	46	0.41	1280	15	100	0.384
Serum KL-6, U/mL	50	0.71	406	54	84	0.043
Honeycomb score	51	0.55	3	85	39	0.891
GGO score	51	0.63	8	92	34	0.137

AUC, area under the receiver-operating characteristic curve; GGO, ground-glass opacity; HO-1, heme oxygenase-1; KL-6, Krebs von den Lungen-6; LDH, lactate dehydrogenase; P/F, partial pressure of oxygen in arterial blood/fraction of inspiratory oxygen; SP-D, surfactant protein-D.

### Primary predictors of 12-week mortality after diagnosis of acute exacerbation

**Figure 3:**
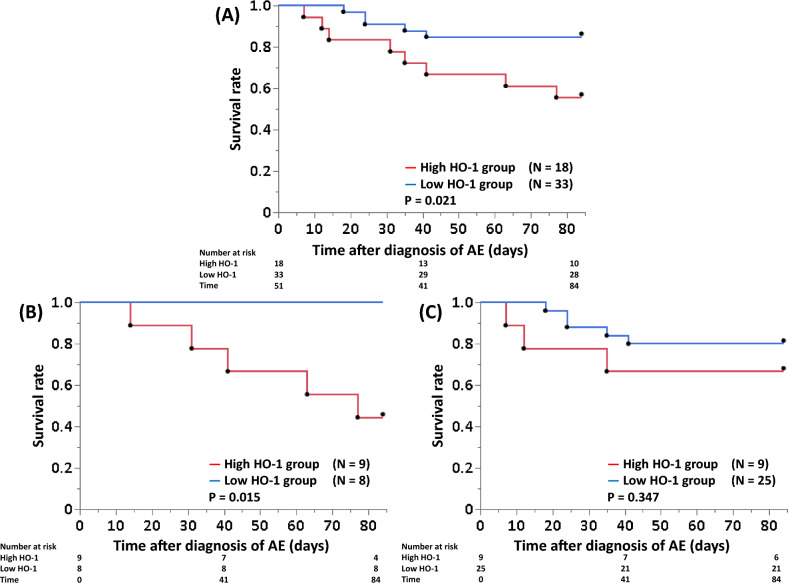
Survival curves according to serum heme oxygenase (HO)-1 level. The best cut-off value of serum HO-1 for predicting 12-week mortality was 41.8 ng/mL, determined by receiver-operating characteristic (ROC) analysis (area under the ROC curve for HO-1 0.71, sensitivity 74%, specificity 62%, *P* = 0.016). Log-rank test showed significant difference between the high and low serum HO-1 groups in the overall patients (*P* = 0.021) (**A**) and in patients with idiopathic pulmonary fibrosis (IPF) (*P* = 0.015) (**B**). There was similar tendency in patients with non-IPF (*P* = 0.347) (C).

We conducted an exhaustive variable selection among models with two explanatory variables based on Uno's c-index calculated from fivefold cross validation. The candidate explanatory variables were age, sex, CCIS, ILD diagnosis, log P/F ratio, log serum HO-1 and log serum KL-6, log serum LDH, GGO score, and honeycomb score (Table 3). Using the best cut-off value of serum HO-1 (41.8 ng/mL) for predicting 12-week mortality, we compared the clinical characteristics (Table 4) and survival curves (Fig. 3) between patients in the high- and low-serum HO-1 groups and found significant difference in serum HO-1 by log-rank test among all enrolled patients and in patients with IPF (*P* = 0.021, Fig. 3A; and 0.015, Fig. 3B, respectively). A similar tendency was observed in patients with non-IPF (*P* = 0.347, Fig. 3C).

**Table 3:** The primary predictors of 12-week mortality selected via the best-subset selection procedure.

Variable	Hazard ratio	95% CI	*P* value
Log serum HO-1	3.116	1.132–8.575	0.028
Log serum KL-6	0.407	0.190–0.875	0.021
C-index (mean value obtained through fivefold cross validation)	0.745		

CI, confidence interval; HO-1, heme oxygenase-1; KL-6, Krebs von den Lungen-6.

**Table 4:** Patient characteristics according to serum HO-1 level.

Characteristic	Low HO-1 group (n = 38)	High HO-1 group (n = 13)	*P* value (Low vs. High )
Age, y	75 (71–80)	75 (69–80)	0.992
Male sex	26 (79)	12 (67)	0.502
CCIS	3 (2–4.5)	3 (2–4)	0.595
Diagnosis of ILDs			
IPF	8 (24)	9 (50)	0.065
Blood biomarkers			
P/F ratio	293 (206–357)	232 (166–262)	0.110
Serum LDH, U/L	261 (208–334)	341 (275–423)	0.007
Serum KL-6, U/L	949 (388–1555)	938 (625–1942)	0.649
Serum SP-D, ng/mL	192 (97–285)	289 (118–421)	0.070
HRCT score (baseline)			
GGO score	8 (5.5–11)	10 (8–16)	0.031
Honeycomb score	4 (1–9)	4 (1.8–8)	0.984
HRCT score (repeat)			
GGO score	5.5 (4–7)	7 (5.5–9)	0.025
Honeycomb score	5.5 (2–9.8)	7 (2–12)	0.612

Data are presented as the median (25th–75th percentiles) or the number (%).

CCIS, Charlson Comorbidity Index score; GGO, ground-glass opacity; HO-1, heme oxygenase-1; HRCT, high-resolution computed tomography; ILDs, interstitial lung diseases; IPF, idiopathic pulmonary fibrosis; KL-6, Krebs von den Lungen-6; LDH, lactate dehydrogenase; P/F ratio, partial pressure of oxygen in arterial blood/fraction of inspiratory oxygen; SP-D, surfactant protein-D.

### Relationship of serum HO-1 to progression of honeycomb and GGO lesions

**Figure 4:**
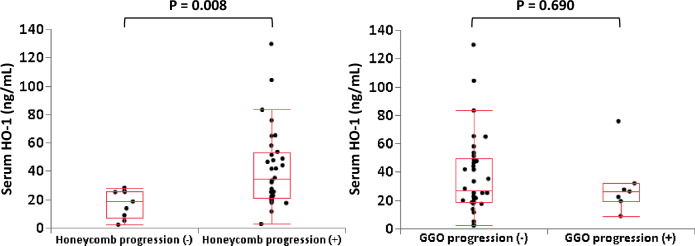
Serum heme oxygenase (HO)-1 according to progression and non-progression of honeycombing and ground glass opacity (GGO) lesions. The baseline serum HO-1 with honeycomb progression ( +) group was higher than that with honeycomb progression ( −) group. On the other hand, there was no significant difference between serum HO-1 with GGO progression ( +) and progression ( −) groups.

Figure 4 summarizes the relationship between serum HO-1 and progression of honeycomb and GGO lesions. Repeat HRCT was obtained in 41/51 patients. The median duration from baseline CT to repeat HRCT was 36 [25–67] days. Based on the absence or presence of progression of honeycombing, these 41 patients were divided into honeycomb progression (–) and ( +) groups. Baseline serum HO-1 was higher in the honeycomb progression ( +) group than in the honeycomb progression (–) group (34.2 [21.1–53.0] ng/mL and 18.5 [7.0–25.7] ng/mL, respectively).

Based on the absence or presence of progression of GGO, there was no significant difference in baseline serum HO-1 between the GGO progression ( +) and progression (–) groups (26.2 [19.4–32.0] ng/mL and 26.9 [18.5–49.4] ng/mL, respectively).

## Discussion

ILDs comprise a group of lung disorders characterized by various levels of inflammation and fibrosis. The disease activity, clinical course, and long-term prognosis are diverse among patients because of the wide range of pathological patterns^25^. Because it is difficult to obtain tissue samples for histological evaluation in patients due to acute respiratory failure, it is necessary to develop a non-invasive biomarker for assessing disease prognosis that can be measured simply and repeatedly. Furthermore, a really important biomarker should alter clinicians’ decision by leading to early diagnosis, providing information regarding disease activity or the need for treatment modification. Particularly in the field of IPF, various biomarkers related to the mechanistic pathway such as alveolar dysfunction (MUC5B, telomerase), immune dysregulation (monocyte, heat shock protein 70, S100A12), extracellular matrix remodelling (collagen degradation biomarkers) have been proposed, however there is a need for high-quality, implementation research to bring these biomarkers into daily clinical practice^26^. As a new biomarker candidate for ILD, we have previously demonstrated that evaluating the degree of oxidative stress by measuring HO-1 in the peripheral blood known as a rate-limiting enzyme in heme catabolism with antioxidative activities was useful for assessing disease activities and predicting prognosis in patients with such as ARDS, COVID-19, and acute exacerbation of ILDs^22,27–30^. In the present study, compared to commonly used clinical parameters such as blood biomarkers and HRCT findings, serum HO-1 proved useful for diagnosing the acute exacerbation of ILD and predicting the short-term prognosis of acute exacerbations. Also, the present findings contribute to our knowledge regarding the relationship between serum HO-1 and subsequent changes in pulmonary fibrosis.

The HO-1 system with these products such as carbon monoxide, iron, and bilirubin represents a powerful tissue protective system that regulates important biological processes, including inflammation, fibrosis, and angiogenesis^31^. Increased serum HO-1 has been reported to correlate with the severity of oxidative stress to the lung^21,22,30^. In the present study, serum HO-1 was significantly higher in acute exacerbation of IPF compared with that in acute exacerbation of IIPs other than IPF or CTD-ILD. Also, serum LDH, which is a known marker of pulmonary cellular damage, was higher in the acute exacerbation of IPF group than in the non-IPF group and mortality within 12 weeks was relatively higher in the former group^32^. Therefore, oxidative stress was thought to be potentially stronger in patients with IPF than in patients with other ILD. As shown in Figs. 1 and 4, the degree of oxidative stress related to acute exacerbation is different in each ILD subtype, suggesting that control of oxidative stress may be an important therapeutic target rather than the underlying ILD.

Increased levels of HO-1 in lung cells and blood to enable prediction of not only the degree of inflammation in the acute exacerbation phase but also of future progression of lung fibrosis. Indeed, in the present study, baseline serum HO-1 was higher in patients who had progression of honeycombing between the baseline and repeat HRCTs than in those without progression. Circulating monocytes have been reported to promote and predict IPF progression^33,34^. Scott et al., by performing cell deconvolution analysis of transcriptome data, reported an unexpected finding of an association between absolute and relative numbers of circulating monocytes and survival in patients with IPF^34^. Circulating monocytes which migrate to the lung are differentiated uncommitted macrophages (M0), and they are broadly polarized to pro-inflammatory M1 macrophages and anti-inflammatory M2 macrophages that highly express HO-1^35–37^. The interaction between M1 and M2 macrophages is reported to be closely correlated with disease progression of acute exacerbation of ILD^36–40^. The M2 macrophages differentiate in response to interleukin (IL)-4, IL-10, and IL-13, and produce large amounts of tissue growth factor-β1 that results in extracellular matrix deposition, fibroblast activation, and cell death after M1 macrophage activation^36,41,42^. It is necessary to perform further clinical and molecular validation to evaluate the mechanism between increased HO-1 protein level and fibrosis formation.

We speculate that serum HO-1 may serve as a useful biomarker for evaluating the severity of oxidative stress to the lung and for predicting 12-week mortality and future fibrosis formation. However, the research described here has several limitations. First, this was a single-centre study with a small number of patients. It is necessary to expand this work in a multi-centre prospective study to evaluate the reproducibility of the present results. Second, we did not perform adequate histological examination due to patients’ severe hypoxemia. It is necessary to identify the origin of HO-1 expression in the lung and to verify the relationship between the degree of expression and the serum HO-1 level. Third, although we have previously reported that elevation of HO-1 induced lethal fibrosis in acute exacerbation of IPF triggered by COVID-19, it is necessary to perform a molecular pathological study regarding the in vivo dynamics of serum HO-1 elevation and pro-fibrotic cytokines such as tissue growth factor-β1^43^.

## Conclusions

Serum HO-1 measurement could be useful for evaluating disease mortality and morbidity of patients with acute exacerbation of ILDs. Also, it is necessary to perform further clinical and molecular validation to evaluate the mechanism between increased HO-1 protein level and fibrosis formation.

## Methods

### Study location and enrolled patients

Enrolled in this study were newly diagnosed and untreated patients with ILD who were admitted to hospital with acute exacerbation between 2011 and 2021. Data including medical history, comorbidities calculated as CCIS, results of blood biomarkers, HRCT findings, treatment, and 12-week mortality were extracted from patients’ medical records^23^. The diagnosis of IPF and IIPs was based on established criteria^44,45^. The diagnosis of CTD-ILD was confirmed by physical findings, serological testing, and HRCT findings that were consistent with ILD. Chronic hypersensitivity pneumonia was diagnosed based on previously established criteria^46^. Acute exacerbation was defined as significant respiratory deterioration including clinical worsening of dyspnoea, hypoxemia, or the worsening or severe impairment of gas exchange characterized by new bilateral GGO /consolidation superimposed on a background pattern consistent with an ILD pattern not fully explained by cardiac failure or fluid overload^47,48^. We ruled out infectious pneumonia based on sputum and blood cultures or clinical evidence that antimicrobials were ineffective. Also, COVID-19 triggered acute exacerbation was ruled out using real-time reverse transcription quantitative polymerase chain reaction on nucleic acids extracted from nasopharyngeal and pharyngeal swab samples at the diagnosis of acute exacerbation. All procedures were performed in accordance with relevant guidelines and regulations. The details of the method followed our previous report^15^.

### HRCT scoring

The HRCT findings were evaluated using the semiquantitative scoring method described by Ooi et al.^24^ The lungs were divided into six distinct zones, three on each side. GGO and honeycombing on HRCT were then scored based on the percentage of disease extent in each of the six zones. The global score was calculated by adding the scores for each abnormality in all lobes. HRCT was performed at the diagnosis of AE and at follow-up (median duration from baseline CT to repeat CT was 36 days), and each scan was independently assessed by three pulmonologists (HY, TY, and MK (each with at least 10 years’ experience).

### Serum HO-1 and other blood biomarker measurements

Serum HO-1 levels were measured at the time of acute exacerbation diagnosis using the IMMUNOSET HO-1 (human) ELISA development set (Enzo, Farmingdale, NY, USA). Other blood samples including P/F ratio, LDH (normal < 225 U/L), SP-D (normal < 110 ng/mL), and KL-6 (normal < 500 U/mL) were obtained at the same time as serum HO-1 measurement. The details of the HO-1 ELISA method have been described previously^22^. Assay validation was performed based on the reproducibility of this ELISA standard curve for serum HO-1, the intra- and inter-assay tests, and the percentage recovery test. It was confirmed that all results were acceptable. The serum HO-1 of normal controls was measured from 19 biobank samples from healthy volunteers before 2019.

### Statistical analysis

Data were statistically analysed using JMP12 (SAS Institute, Cary, NC) and R software, version 4.2.1 (The R Foundation for Statistical Computing, Vienna, Austria), and are presented as the median (25th–75th percentiles) or the number (%). Groups were compared using chi-square test and Wilcoxon rank-sum test. Non-parametric Spearman’s rank correlation coefficient was calculated to assess the correlation of serum HO-1 level with other clinical parameters. To determine the primary predictors of 12-week mortality, the best-subset selection procedure from candidates or uni- and multi-variable analyses were performed. A time-dependent ROC curve analysis was performed to determine the most suitable cut-off level of serum HO-1 for predicting 12-week mortality. Kaplan–Meier curves were used to compare 12-week mortality between the high and low HO-1 groups. Log-rank testing was also performed with strata based on the identified predictors. Values of *P* < 0.05 were considered significant.

### Study approval

All participants provided informed consent prior to participation in this research. All aspects of the study were approved by the Institutional Review Board of Yokohama City University Graduate School of Medicine (approval number B170900025). The authors conducted this research in full accordance with the Declaration of Helsinki.

## Data Availability

All data generated or analyzed during this study are included in this published article.

## References

[1] Hyzy R (2007). Acute exacerbation of idiopathic pulmonary fibrosis. Chest.

[2] Kim DS (2006). Acute exacerbation of idiopathic pulmonary fibrosis: frequency and clinical features. Eur. Respir. J..

[3] Usui Y (2013). A cohort study of mortality predictors in patients with acute exacerbation of chronic fibrosing interstitial pneumonia. BMJ Open.

[4] Tachikawa R (2012). Clinical features and outcome of acute exacerbation of interstitial pneumonia: collagen vascular diseases-related versus idiopathic. Respiration.

[5] Oda K (2014). Autopsy analyses in acute exacerbation of idiopathic pulmonary fibrosis. Respir. Res..

[6] Fujii H (2023). ILD-GAP combined with the Charlson Comorbidity Index score (ILD-GAPC) as a prognostic prediction model in patients with interstitial lung disease. Can. Respir. J..

[7] Murohashi K (2019). Clinical significance of Charlson comorbidity index as a prognostic parameter for patients with acute or subacute idiopathic interstitial pneumonias and acute exacerbation of collagen vascular diseases-related interstitial pneumonia. J. Thorac. Dis..

[8] Hosseinzadeh A (2018). Oxidative/nitrosative stress, autophagy and apoptosis as therapeutic targets of melatonin in idiopathic pulmonary fibrosis. Expert Opin. Ther. Targets.

[9] Cameli P (2020). Oxidant/antioxidant disequilibrium in idiopathic pulmonary fibrosis pathogenesis. Inflammation.

[10] Cameli P (2019). Alveolar concentration of nitric oxide as a prognostic biomarker in idiopathic pulmonary fibrosis. Nitric Oxide.

[11] Walters DM, Cho H, Kleeberger SR (2008). Oxidative stress and antioxidants in the pathogenesis of pulmonary fibrosis: a potential role of Nrf2. Antioxid. Redox Signal..

[12] Chung-man, Ho J. *et al*. Differential expression of manganese superoxide dismutase and catalase in lung cancer. *Cancer. Res.* **61**, 8578–8585 (2001). 11731445

[13] Smith LJ (1997). Reduced superoxide dismutase in lung cells of patients with asthma. Free. Radic. Biol. Med..

[14] Yildiz L (2002). The changes of superoxide dismutase, catalase and glutathione peroxidase activities in erythrocytes of active and passive smokers. Clin. Chem. Lab. Med..

[15] Vlahos R, Bozinovski S (2013). Glutathione peroxidase-1 as a novel therapeutic target for COPD. Redox. Rep..

[16] Ambade VN (2015). Diagnostic utility of biomarkers in COPD. Respir. Care..

[17] Yousefi-Manesh H (2022). Protective effect of dapsone against bleomycin-induced lung fibrosis in rat. Exp. Mol. Pathol..

[18] Choi AMK, Alam J (1996). Heme oxygenase-1: function, regulation, and implication of a novel stress-inducible protein in oxidant-induced lung injury. Am. J. Respir. Cell. Mol. Biol..

[19] Mumby S (2004). Lung heme oxygenase-1 is elevated in acute respiratory distress syndrome. Crit. Care Med..

[20] Lakari E (2001). Expression and regulation of hemeoxygenase 1 in healthy human lung and interstitial lung disorders. Hum. Pathol..

[21] Kata Y (2022). Assessment of diagnostic utility of serum hemeoxygenase-1 measurement for acute exacerbation of interstitial pneumonias. Sci. Rep..

[22] Hara Y (2018). ELISA development for serum hemeoxygenase-1 and its application to patients with acute respiratory distress syndrome. Can. Respir. J..

[23] Charlson ME, Pompei P, Ales KL, MacKenzie CR (1987). A new method of classifying prognostic comorbidity in longitudinal studies: development and validation. J. Chronic Dis..

[24] Ooi GC (2003). Interstitial lung disease in systemic sclerosis. Acta Radiol..

[25] Arai T (2016). Heterogeneity of incidence and outcome of acute exacerbation in idiopathic interstitial pneumonia. Respirology.

[26] Karampitsakos T (2023). Precision medicine advances in idiopathic pulmonary fibrosis. EBioMedicine..

[27] Sato T (2006). Heme oxygenase-1, a potential biomarker of chronic silicosis, attenuates silica-induced lung injury. Am. J. Respir. Crit. Care Med..

[28] Nakashima K (2018). Regulatory role of heme oxygenase-1 in silica-induced lung injury. Respir. Res..

[29] Hara Y (2022). Heme oxygenase-1 as an important predictor of the severity of COVID-19. PLOS One.

[30] Murohashi K (2018). Clinical significance of serum hemeoxygenase-1 as a new biomarker for the patients with interstitial pneumonia. Can. Respir. J..

[31] Loboda A (2016). Role of Nrf2/HO-1 system in development, oxidative stress response and diseases: an evolutionarily conserved mechanism. Cell. Mol. Life Sci..

[32] DeRemee RA (1968). Serum lactic dehydrogenase activity and diffuse interstitial pneumonitis. JAMA.

[33] Michael K (2021). Monocyte count as a prognostic biomarker in patients with idiopathic pulmonary fibrosis. Am J Respir Crit Care Med.

[34] Scott MKD (2019). Increased monocyte count as a cellular biomarker for poor outcomes in fibrotic diseases: a retrospective, multicentre cohort study. Lancet Respir. Med.

[35] Gordon S (2003). Alternative activation of macrophages. Nat. Rev. Immunol..

[36] Boyle JJ (2009). Coronary intraplaque hemorrhage evokes a novel atheroprotective macrophage phenotype. Am. J. Pathol..

[37] Kadl A (2010). Identification of a novel macrophage phenotype that develops in response to atherogenic phospholipids via Nrf2. Circ. Res..

[38] Nouno T (2019). Elevation of pulmonary CD163+ and CD204+ macrophages is associated with the clinical course of idiopathic pulmonary fibrosis patients. J. Thorac. Dis..

[39] Yamashita M (2018). Distinct profiles of CD163-positive macrophages in idiopathic interstitial pneumonias. J. Immunol. Res..

[40] Schupp JC (2015). Macrophage activation in acute exacerbation of idiopathic pulmonary fibrosis. PLOS One.

[41] Murray LA (2011). TGF-beta driven lung fibrosis is macrophage dependent and blocked by serum amyloid *P*. Int. J. Biochem. Cell. Biol..

[42] Pohlers D (2009). TGF-beta and fibrosis in different organs - molecular pathway imprints. Biochim. Biophys. Acta.

[43] Hara Y (2022). Clinical importance of serum heme oxygenase-1 measurement in patients with acute exacerbation of idiopathic pulmonary fibrosis triggered by coronavirus disease 2019. Respir. Med. Case Rep..

[44] Raghu, G. *et al*. Diagnosis of idiopathic pulmonary fibrosis. An Official ATS/ERS/JRS/ALAT Clinical Practice Guideline. *Am. J. Respir. Care Med.* **198**, 44–68 (2018). 10.1164/rccm.201807-1255ST 30168753

[45] Travis WD (2013). An official American Thoracic Society/European Respiratory Society statement: Update of the international multidisciplinary classification of the idiopathic interstitial pneumonias. Am. J. Respir. Care Med..

[46] Lacasse Y (2003). Clinical diagnosis of hypersensitivity pneumonitis. Am. J. Respir. Crit. Care. Med..

[47] Collard, H. R. *et al.* Acute exacerbation of idiopathic pulmonary fibrosis. An International Working Group Report. *Am. J. Respir. Care Med.* **194**, 265–275 (2016). 10.1164/rccm.201604-0801CI 27299520

[48] Park IN (2007). Acute exacerbation of interstitial pneumonia other than idiopathic pulmonary fibrosis. Chest.

